# Mediating role of disease acceptance in the relationship between social support and adherence to treatment in patients with type 2 diabetes

**DOI:** 10.1007/s10865-025-00567-w

**Published:** 2025-04-05

**Authors:** Gülyeter Erdoğan Yüce, Heja Yıldırım

**Affiliations:** 1 Faculty of Health Sciences, Aksaray University, Aksaray, 68100 Turkey; 2 Institute of Health Sciences, Aksaray University, Aksaray, 68100 Turkey

**Keywords:** Disease acceptance, Social support, Treatment adherence, Type 2 diabetes

## Abstract

Type 2 diabetes is a widespread, chronic metabolic disease with significant consequences for public health. Poor adherence to treatment in type 2 diabetes patients is associated with inadequate glycemic control, increased medical costs, increased use of healthcare services, and increased mortality. Understanding how social support and disease acceptance influence treatment adherence can inform more effective diabetes management strategies. This study investigated the relationship between social support, disease acceptance, and treatment adherence in adults with type 2 diabetes. This cross-sectional correlational study was conducted on 332 individuals with type 2 diabetes in Türkiye. Data were collected using a patient information form, Acceptance of Illness Scale, Multidimensional Perceived Social Support Scale, and type 2 Diabetes Treatment Adherence Scale. Relationships between variables were analyzed using Pearson correlation coefficients, linear regression, and mediation analysis. The regression analysis indicated significant effects of social support on treatment adherence (β=-0.312, *p* < 0.001) and disease acceptance (β = 0.267, *p* < 0.001) and of disease acceptance on treatment adherence (β=-0.378, *p* < 0.001). Additionally, disease acceptance partially mediated the relationship between social support and treatment adherence (β=-0.101, %95 Cl [-0.154, -0.055]). The findings showed that high levels of social support and illness acceptance can improve treatment adherence and that social support may also indirectly enhance treatment adherence by promoting illness acceptance.

## Introduction

Type 2 diabetes is a widespread, chronic metabolic disease with significant consequences for public health (Sun et al., 2022). The main goal of diabetes treatment is to prevent or delay diabetes-related complications, reduce mortality and increase the quality of life by providing glycemic control (Kayar et al., 2017). In addition to regular medication, the treatment brings many lifestyle changes, such as self-monitoring blood sugar, dietary restrictions, weight control, exercise, regular foot care and eye examinations (Arı & Özdelikara, 2022; Sharma et al., 2014; Taşkın Yılmaz et al., 2019). Effective diabetes management can be achieved by adhering with all aspects of the treatment (Arı & Özdelikara, 2022). WHO defines adherence to treatment as taking the prescribed drugs at the appropriate time and in appropriate doses, as well as accepting and applying the recommendations about the patient’s health, such as participation in controls, diet and lifestyle changes (World Health Organization, 2003). Poor adherence to treatment in type 2 diabetes patients is associated with inadequate glycemic control, increased medical costs, increased use of healthcare services, and increased mortality (Lin et al., 2017; Polonsky & Henry, 2016). Treatment adherence in diabetes is a complex behavior influenced by factors related to the patient, providers, and healthcare systems throughout the care process (Barasa Masaba & Mmusi-Phetoe, 2021). Previous studies have shown that treatment adherence of individuals with diabetes is affected by many factors such as treatment complexity, disease duration, care delivery, patient’s age, sex, self-esteem, disease acceptance, self-efficacy and stress level, quality of the relationship between patients and care providers, environmental factors and social support (Barasa Masaba & Mmusi-Phetoe, 2021; Polonsky & Henry, 2016; Shahabi et al., 2023; Świątoniowska-Lonc et al., 2021).

In this context, the Self-Determination Theory provides an important framework for understanding the diabetes management process, which involves complex health behaviours to be maintained in the long term and for addressing treatment adherence in individuals with diabetes (Okati-Aliabad et al., 2024). According to this theory, individuals’ motivation to sustain behaviors is divided into two categories: autonomous and controlled. Autonomous motivation includes identified regulation, integrated regulation, and intrinsic motivation, whereas controlled motivation encompasses introjected and external regulations. Controlled motivation refers to regulating behaviors through external sources, such as rewards and approval, or internal pressures, such as guilt and shame (Patrick & Williams, 2012; Ryan & Deci, 2000; Schmidt et al., 2020).

Self-Determination Theory emphasizes the significant role of an individual’s social environment in motivating behavior change (De Man et al., 2020). Although the primary responsibility for self-management in diabetes belongs to the individual, the support provided by social networks can make it easier for the individual to fulfil the duties required to maintain blood glucose levels (Bouldin et al., 2017; Onyango et al., 2023; Smalls et al., 2015; Świątoniowska-Lonc et al., 2021). Lack of social support has been identified as a factor restricting treatment adherence in patients with type 2 diabetes (Bouldin et al., 2017; Smalls et al., 2015; Świątoniowska-Lonc et al., 2021). Social support enhances motivation, strengthens the sense of competence, and contributes to developing necessary skills in individuals with diabetes, directly or through reinforcement expectations. Furthermore, social support functions as an environmental resource that facilitates the maintenance of health behaviors (Chen et al., 2022; Misra & Lager, 2008). In this context, social support serves as a crucial source of both controlled and autonomous motivation for individuals with diabetes. While it fosters controlled motivation by providing external reinforcement, such as practical and emotional assistance, it also enhances autonomous motivation by fulfilling psychological needs for competence, relatedness, and autonomy, helping individuals maintain their treatment regimens. Social support enhances an individual’s motivation and supports disease acceptance by fulfilling fundamental psychological needs. Studies conducted with individuals with diabetes have shown a positive relationship between social support and disease acceptance, indicating that individuals with higher levels of social support tend to have higher levels of disease acceptance (Besen & Esen, 2012; Misra & Lager, 2008).

As Self-Determination Theory states, lasting behavior change occurs through internalizing the behavior (Patrick & Williams, 2012; Ryan & Deci, 2000). Accepting their current condition is a prerequisite for individuals to internalize behaviour change. Acceptance of diabetes is defined as the individual’s acceptance of the physical and mental burden of diabetes and its psychosocial impact on their life, with all its positive and negative aspects, without feeling inadequate or dependent (Akturk & Aydinalp, 2018; Schmitt et al., 2014). Disease acceptance refers to a psychological adaptation process in which individuals become more active in their self-care and learn to face the limitations imposed by the disease with optimism (Bertolin et al., 2015). Patients with a high level of disease acceptance can better adapt to necessary lifestyle changes associated with chronic illnesses (Iwanowicz-Palus et al., 2019). Individuals who accept diabetes continue their daily lives more easily with the disease and overcome the individual, family, professional, and social challenges caused by it more effectively (Taşkın Yılmaz et al., 2019). Those who accept the disease tend to internalize their behaviors more effectively and make more lasting changes in diabetes management (Rashidi & Yıldırım, 2024). Previous studies have reported that disease acceptance in individuals with type 2 diabetes is positively associated with treatment adherence and self-efficacy (Akturk & Aydinalp, 2018; Can et al., 2020; Özkaptan et al., 2019). Disease acceptance can be considered a factor that enhances autonomous motivation within the framework of Self-Determination Theory. It may help individuals with diabetes feel competent, independent, and socially connected, thereby contributing to the sustainability of their motivation.

As emphasized within the framework of Self-Determination Theory, disease acceptance and social support represent critical psychosocial variables that influence both behavioral and psychological adaptation processes in treatment adherence among individuals with diabetes. Although previous studies have shown that social support and disease acceptance are associated with diabetes treatment adherence, the dynamic interaction between social support and disease acceptance and its impact on treatment adherence has not been sufficiently investigated. Addressing the direct and indirect relationships between these variables may contribute to developing more effective intervention strategies by the healthcare system and healthcare professionals to enhance treatment adherence among individuals with diabetes. This study seeks to address the identified knowledge gap by exploring the interactions between social support and disease acceptance in the context of adherence to diabetes treatment. Specifically, the study had two primary objectives: (a) to examine the relationships between social support, disease acceptance, and diabetes treatment adherence among adults with type 2 diabetes, and (b) to investigate the mediating role of disease acceptance in the relationship between social support and diabetes treatment adherence. Sociodemographic and clinical variables (such as age, education, income, duration of diabetes, diabetes treatment, and chronic diseases other than diabetes) known to influence treatment adherence in diabetes were included in the study as control variables to isolate their effects. Based on the literature analysis, the following hypotheses were formulated: (i) Social support fosters greater adherence to treatment, (ii) Social support contributes to the acceptance of illness, (iii) Acceptance of illness facilitates improved adherence to treatment, and (iv) Acceptance of illness mediates the relationship between social support and treatment adherence.

## Methods

### Study design, setting and participants

This study employed a cross-sectional correlational design to examine the relationships between social support, disease acceptance, and treatment adherence, with a specific focus on the mediating role of disease acceptance. The research was conducted between February and May 2023 with type 2 diabetes patients receiving outpatient treatment at a 2nd level public hospital in the Central Anatolia region of Türkiye. A convenience sampling method was used in the study. Individuals aged 18 years and older, with a diagnosis of type 2 diabetes for at least one year, undergoing treatment, literate, able to speak and understand Turkish, and with the physical and cognitive capacity to complete the data collection forms were included in the study. Individuals with visual or hearing impairments, pregnant individuals, those with cognitive limitations, those experiencing severe health conditions related to acute diabetes complications, or those with significantly impaired general health that could hinder their ability to participate in the study were excluded. The sample size was calculated using the GPower V3.1.9.7 program (Faul et al., 2009). In the a priori power analysis performed assuming an effect size of 0.20, power (1-β) of 95%, and type I error (α) of 0.05, the minimum sample size for the study was determined as 327 people. A total of 392 individuals were approached, and the study was completed with 332 participants who met the inclusion criteria and agreed to participate, resulting in a participation rate of approximately 84.69%.

### Data collection

Data were collected through face-to-face interviews using the Patient Information Form, the Illness Acceptance Scale, the Multidimensional Perceived Social Support Scale, and the type 2 Diabetes Mellitus Treatment Patient Adherence Scale. The interviews were conducted in a private room at the outpatient clinic and lasted an average of 20 min. Participants were asked to independently read and complete the forms after receiving the necessary explanations, which aimed to encourage honest responses and reduce potential self-presentation bias. During the interviews, the completed forms were reviewed in real time to ensure no responses were missing, and participants were guided to provide any missing information before finalizing the forms. This approach ensured data completeness and consistency.

### Data collection tools

#### Patient information form

This form was designed to collect sociodemographic and clinical information from participants. Sociodemographic characteristics included age (measured in years and categorized into age groups), sex, marital status, education level (literate, primary, high school, university and above), income status (income less than expenses, income equals expenses, income more than expenses), and living arrangements (living with spouse and child, living with spouse only, living alone). Clinical characteristics assessed included the status of receiving structured diabetes education (yes/no), treatment type (oral antidiabetics, oral antidiabetics + insulin, insulin only), and duration of diabetes (measured in years and categorized into ranges). Participants also reported their frequency of blood glucose monitoring (e.g., daily, weekly, when feeling unwell, never) and chronic diseases other than diabetes (yes/no). This form provided detailed data on variables relevant to understanding participants’ diabetes management and treatment adherence.

#### Acceptance of illness scale

The scale developed by Felton and Revenson (1984) determines the patient’s degree of disease acceptance (Felton et al., 1984). It was adapted by Besen and Esen (2011) for patients with type 2 diabetes in the Turkish population. The scale evaluates the limitations and negative emotions caused by the disease and the feelings of worthiness and acceptance. The scale consists of 8 items, including statements such as ‘I have difficulty adjusting to the limitations imposed by my illness’ and ‘Due to my health condition, I miss doing the things I enjoy the most’ The scale uses a 5-point Likert format, where participants are asked to rate how much they agree with the statements on a scale ranging from ‘Strongly Agree’ (1) to ‘Strongly Disagree’ (5). Only the 6th item is reverse-scored in the scale. The lowest eight and the highest 40 points are taken from the scale. Lower scores on the scale indicate lower levels of illness acceptance, while higher scores reflect better illness acceptance. The Cronbach Alpha value of the Turkish version of the scale is 0.79 (Besen & Esen, 2011). In this study, the Cronbach Alpha value was determined as 0.85.

#### Multidimensional scale of perceived social support

The scale developed by Zimet et al. (1988) aims to determine the sources of social support perceived by individuals. The scale consists of 12 items, including statements such as ‘My family tries to help me’, ‘I can talk about my problems with my friends’, and ‘There is a special person around when I am in need’. Participants are asked to rate their level of agreement with each statement using a 7-point Likert scale, where response options range from ‘Definitely No’ (1) to ‘Definitely Yes’ (7). The scale consists of three sub-dimensions: family, friend and a particular person. There are four items in each sub-dimension in the scale. Sub-dimension scores are obtained by summing the scores of the items in the sub-dimensions, and the scale’s total score is obtained by summing all the item scores. A total score ranging from a minimum of 12 to a maximum of 84 can be obtained from the scale. Higher sub-dimensions and total scores mean higher perceived social support. The Cronbach’s alpha reliability coefficient for the sub-dimensions and total scale adapted to Turkish was between 0.80 and 0.95 (Eker et al., 2001). In this study, Cronbach’s alpha values were determined to be between 0.80 and 0.94.

#### Patient adherence scale to type 2 diabetes mellitus treatment

The scale developed by Demirtaş and Akbayrak (2017) is designed to measure adherence to diabetes treatment. The scale consists of 30 items, including statements such as ‘I regularly measure and monitor my blood sugar levels,’ ‘I feel my blood sugar rising,’ and ‘I exercise regularly in summer and winter as recommended.’ It includes 13 positive behavior items and 17 negative behavior items. Participants rate the items using a 5-point Likert scale with response options: ‘Strongly Agree,’ ‘Agree,’ ‘Partially Agree,’ ‘Disagree,’ and ‘Strongly Disagree.’ Positive items are scored from 1 to 5, while the scores for negative items are reversed. The scale has seven sub-dimensions: attitudes and emotional factors, knowledge and personal factors, lifestyle changes, feelings of anger, adaptive emotions and behaviors, diet negotiation, and denial. The total scale score ranges from 30 to 150, with higher scores reflecting poorer treatment adherence. Total scores are interpreted as follows: scores between 30 and 54 indicate ‘good adherence,’ scores between 55 and 125 indicate ‘moderate adherence,’ and scores between 126 and 150 indicate ‘poor adherence’. For the original scale, Cronbach’s alpha was 0.77 (Demirtaş & Akbayrak, 2017). In this study, Cronbach’s alpha was determined to be 0.75.

### Statistical analysis

Data were analyzed using IBM SPSS Version 23.0 and SPSS Process Macro Version 4.2 programs. Statistical significance was accepted as *p* < 0.05. Descriptive statistics were summarized by number, percentage, mean, and standard deviation. The conformity of the data to the normal distribution was evaluated by kurtosis-skewness. Independent samples t-test and one-way ANOVA (Post hoc test Bonferroni) were used to determine the differences in scale scores according to sociodemographic and clinical characteristics. The Pearson correlation coefficient was used to test the relationship between social support, acceptance of illness, and adherence to diabetes treatment. The bootstrap method evaluated Mediation analysis with SPSS Process Macro Version 4.2 (Hayes, 2022). The mediating role of acceptance of illness (Mi) in the effect of social support (X) on adherence to diabetes treatment (Y) was examined using PROCESS model 4 (Hayes, 2022). Age, education, income, duration of diabetes, diabetes treatment type, and the presence of chronic diseases other than diabetes were included as covariates (Co) to control for demographic and clinical factors in the analysis. The analysis used 5.000 samples with the bootstrap method to estimate the mediator effect of disease admission with a 95% confidence interval (CI); the effect was considered significant if the CI did not contain zero (Hayes, 2022).

### Ethical consideration

The study complied with the principles of the Declaration of Helsinki. Permission to carry out the study was obtained from the relevant Clinical Research Ethics Committee and the institution where the study was conducted. All participants were informed about the research before the data collection process, and their informed consent was obtained.

## Results

The participants had a mean age of 60.75 ± 11.83 years (range: 19–85 years), with 43.4% aged 65 years and older. Of the participants, 56.6% were women, 86.1% were married, and 55.1% were primary school graduates. Regarding clinical characteristics, 29.8% had been diagnosed with diabetes for 1–5 years, 72.0% had other chronic diseases besides diabetes, 47.3% were using oral antidiabetic drugs, and 66.0% had not received any structured education about diabetes (Table 1).

**Table 1:** Sociodemographic and clinical characteristics of the participants (*n* = 332)

Sociodemographic characteristics	Mean ± SS or *n* (%)	Clinical characteristics	Mean ± SS or *n* (%)
**Age (years)**	60.75 ± 11.83	**Status of receiving structured diabetes education**	
**Age groups**		Yes	113 (34.0)
Under 65 years old	188 (56.6)	No	219 (66.0)
65 years and older	144 (43.4)	**Treatment of diabetes**	
**Sex**		Oral antidiabetic	157 (47.3)
Male	144 (43.4)	Oral antidiabetic + insulin	96 (28.9)
Female	188 (56.6)	Insulin	79 (23.8)
**Marital status**		**Duration of diabetes**	
Married	286 (86.1)	1–5 years	99 (29.8)
Single	46 (13.9)	6–10 years	89 (26.8)
**Education**		11–15 years	62 (18.7)
Literate	72 (21.7)	16–20 years	40 (12.0)
Primary school	183 (55.1)	21 and over years	42 (12.7)
High school	41 (12.3)	**Frequency of blood glucose monitoring at home**	
University and above	36 (10.8)	Every day	95 (28.6)
**Income**		Around the day	34 (10.2)
Income less than expense	51 (15.4)	Weekly	49 (14.8)
Income equals expense	214 (64.5)	When feeling unwell	114 (34.3)
Income is more than expense	67 (20.2)	Never	25 (7.5)
**Person(s) living with**		I lack a glucose meter	15 (4.5)
Spouse and Child	136 (41.0)	**Chronic diseases other than diabetes**	
With his wife/ her husband	121 (36.4)		
With his/her children	37 (11.1)	Yes	239 (72.0)
Alone	38 (11.4)	No	93 (28.0)

The mean acceptance of illness scale score was 27.47 ± 7.71, the mean score on the social support scale was 61.90 ± 18.26, and the mean score on the adherence scale to diabetes treatment was 79.56 ± 12.63 (Table 2).

**Table 2:** Mean scores of participants on disease acceptance, social support and treatment adherence (*n* = 332)

Variables	Mean ± SS	Min-Max
**Acceptance of illness scale**	27.47 ± 7.71	8.00–40.00
**Multidimensional scale of perceived social support**		
Family	7.42 ± 7.18	4.00–28.00
Friend	19.87 ± 6.66	4.00–28.00
A special person	22.61 ± 5.71	4.00–28.00
Total score	61.90 ± 18.26	12.00–84.00
**Patient adherence scale to Type 2 diabetes mellitus treatment**		
Attitude and emotional factors	23.32 ± 5.47	8.00–38.00
Knowledge and personal factors	14.53 ± 4.07	6.00–27.00
Lifestyle changes	9.19 ± 2.96	3.00–15.00
Feelings of anger	8.09 ± 2.55	3.00–15.00
Adaptive emotions and behaviors	7.16 ± 2.54	4.00–14.00
Diet negotiation	9.33 ± 2.38	3.00–15.00
Denial	7.91 ± 2.70	3.00–15.00
Total score	79.56 ± 12.63	36.00-117.00

Acceptance of Illness Scale mean scores showed a statistically significant difference in age, education, income, duration of diabetes, diabetes treatment and presence of diseases other than diabetes (*p* < 0.05). Acceptance of illness was higher among participants under the age of 65, those with income equal to expenses, those using oral antidiabetics, and those without additional chronic diseases. At the same time, it was lower among participants with a diabetes duration of 21 years or more and those who were literate compared to other groups. A significant difference was determined in the mean scores of the Multidimensional Scale of Perceived Social Support according to the variables of education, income, diabetes treatment and presence of diseases other than diabetes (*p* < 0.05). Perceived social support was found to be lower among participants who were literate or primary school graduates compared to university graduates, those with lower income compared to other income groups, those using both oral antidiabetics and insulin compared to those using only oral antidiabetics, and those with additional chronic diseases compared to those without. It was found that the mean score of the Patient Adherence Scale to Type 2 Diabetes Mellitus Treatment differed statistically according to education and the presence of additional chronic disease (*p* < 0.05). It was determined that participants with a university degree or higher had better treatment adherence than those who were literate, and those without additional chronic diseases had better adherence than those with chronic diseases. (Table 3).

**Table 3:** Distribution of mean scores of disease acceptance, social support and treatment adherence according to sociodemographic and clinical characteristics (*n* = 332)

Sociodemographic and clinical characteristics	Acceptance of illness scale		Multidimensional scale of perceived social support		Patient adherence scale to Type 2 diabetes mellitus treatment	
	Mean ± SS	*p*	Mean ± SS	*p*	Mean ± SS	*p*
**Sex** ^†^						
Woman	28.84 ± 7.86	0.088	61.65 ± 18.80	0.776	79.78 ± 11.82	0.719
Male	28.29 ± 7.47		62.23 ± 17.58		79.27 ± 13.66	
**Age** ^**†**^						
Under 65 years old	29.09 ± 6.57	**< 0.001**	63.02 ± 18.78	0.203	79.63 ± 12.51	0.902
65 years and older	25.35 ± 8.56		60.45 ± 17.51		79.46 ± 12.83	
**Marital status** ^†^						
Married	27.67 ± 7.33	0.337	62.52 ± 17.73	0.126	62.52 ± 17.73	0.126
Single	26.21 ± 9.77		58.08 ± 21.09		58.08 ± 21.09	
**Education** ^‡^						
Literate (a)	25.19 ± 8.46	**0.027** a < b,c, d	59.20 ± 16.88	**0.026** a, b < d	81.90 ± 10.41	**0.042** d < a
Primary school (b)	27.81 ± 7.50		60.78 ± 19.71		79.49 ± 13.16	
High school (c)	28.39 ± 7.67		65.56 ± 15.69		80.12 ± 11.94	
University and above (d)	29.25 ± 6.48		68.88 ± 13.63		74.58 ± 13.73	
**Income** ^‡^						
Income less than expense (a)	25.92 ± 8.18	**0.006** a, c < b	54.56 ± 21.38	**0.007** a < b, c	81.21 ± 9.75	0.136
Income equals expense (b)	28.46.±7.31		63.53 ± 17.05		78.53 ± 13.11	
Income is more than expense (c)	25.49 ± 8.13		62.29 ± 18.36		81.58 ± 12.80	
**Duration of diabetes** ^‡^						
1–5 years (a)	29.59 ± 6.71	**0.001** e < a	65.25 ± 17.21	0.217	80.35 ± 11.47	0.222
6–10 years (b)	27.19 ± 7.83		59.00 ± 15.63		80.11 ± 12.05	
11–15 years (c)	27.43 ± 7.12		61.01 ± 20.06		78.38 ± 13.94	
16–20 years (d)	26.75 ± 8.79		61.30 ± 20.99		81.90 ± 12.53	
21 years and over (e)	11.80 ± 8.19		62.09 ± 19.86		76.04 ± 14.20	
**Diabetes treatment** ^‡^						
Oral antidiabetic	29.44 ± 6.88	**< 0.001** b, c < a	64.51 ± 16.79	**0.046** b < a	78.57 ± 12.15	0.349
Oral antidiabetic & insulin	25.60 ± 8.40		59.20 ± 19.31		80.92 ± 13.22	
Insulin	25.82 ± 7.54		60.01 ± 19.24		79.86 ± 12.83	
**Chronic diseases other than diabetes** ^†^						
Yes	26.61 ± 8.02	**< 0.001**	60.07 ± 18.19	**0.003**	80.47 ± 12.60	**0.034**
No	29.66 ± 6.38		66.61 ± 17.68		77.21 ± 12.48	
**Status of receiving structured diabetes education** ^†^						
Yes	27.96 ± 6.91	0.381	63.62 ± 17.16	0.219	78.15 ± 14.35	0.172
No	27.21 ± 8.10		61.02 ± 18.77		80.29 ± 11.62	

^†^ Independent sample t-test ^‡^ One-way ANOVA test

There was a moderately positive and statistically significant relationship between the scores of the Acceptance of Illness Scale and the Multidimensional Scale of Perceived Social Support (*r* = 0.330, *p* < 0.01). The Patient Adherence Scale to Type 2 Diabetes Mellitus Treatment showed moderate negative correlations with both the Multidimensional Scale of Perceived Social Support (*r* = -0.436, *p* < 0.01) and the Acceptance of Illness Scale (*r* = -0.449, *p* < 0.01) (Table 4).

**Table 4:** The relationship between participants’ levels of disease acceptance, social support, and treatment adherence (*n* = 332)

Variables		1	2	3	4	5	6	7	8	9	10	11	12
Acceptance of illness scale		1											
Multidimensional scale of perceived social support	Family	0.302^**^	1										
	A special person	0.306^**^	0.752^**^	1									
	Friend	0.317^**^	0.869^**^	0.786^**^	1								
	Total score	0.330^**^	0.946^**^	0.896^**^	0.953^**^	1							
Patient adherence scale to Type 2 diabetes mellitus treatment	Attitude and emotional factors	-0.514^**^	-0.436^**^	-0.397^**^	-0.406^**^	-0.444^**^	1						
	Information and personal factors	-0.211^**^	-0.212^**^	-0.239^**^	-0.272^**^	-0.257^**^	0.322^**^	1					
	Lifestyle change	-0.363^**^	-0.267^**^	-0.239^**^	-0.253^**^	-0.272^**^	0.411^**^	0.487^**^	1				
	Feelings of anger	-0.334^**^	-0.329^**^	-0.345^**^	-0.334^**^	-0.359^**^	0.678^**^	0.213^**^	0.212^**^	1			
	Emotions and behaviours appropriate to harmony	-0.161^**^	-0.112^*^	-0.204^**^	-0.171^**^	-0.170^**^	0.132^*^	0.448^**^	0.221^**^	0.062	1		
	Diet bargaining	-0.133^*^	-0.044	-0.102	-0.072	-0.075	0.094	-0.039	-0.099	0.108^*^	-0.016	1	
	A sense of denial	0.246^**^	0.118^*^	0.076	0.121^*^	0.114^*^	-0.176^**^	-0.011	-0.149^**^	-0.046	0.000	0.167^**^	1
	Total Score	-0.449^**^	-0.392^**^	-0.419^**^	-0.413^**^	-0.436^**^	0.777^**^	0.700^**^	0.607^**^	0.637^**^	0.465^**^	0.248^**^	0.121^*^

*p < 0.05 **p < 0.01

Table 5 presents the results of the mediation analysis examining the effect of social support on treatment adherence through disease acceptance, as well as the influence of demographic and clinical factors such as age, education level, income status, duration of diabetes, treatment type, and the presence of additional chronic diseases. According to the results, social support significantly increases disease acceptance (β = 0.267, *p* < 0.001). Higher social support decreases treatment adherence scores (β = -0.413, *p* < 0.001), indicating that increased social support leads to improved treatment adherence. Similarly, higher disease acceptance positively impacts treatment adherence (β = -0.378, *p* < 0.001). When demographic and clinical factors are considered, increased age (β = -0.112, *p* < 0.05) and longer duration of diabetes (β = -0.157, *p* < 0.01) are associated with better treatment adherence. Additionally, individuals whose income equals their expenses demonstrate better adherence to treatment (β = -0.133, *p* < 0.05). Social support, when combined with education level (literacy) and duration of diabetes, explains 23.6% of the variance in treatment adherence. When social support and disease acceptance are analyzed alongside age and duration of diabetes, they collectively explain 34.7% of the variance in treatment adherence. These findings highlight that increased social support and disease acceptance significantly improve treatment adherence and that demographic and clinical factors influence this relationship.

**Table 5:** Mediation effect of disease acceptance between social support and treatment adherence, adjusted for demographic and clinical covariates

Predictors	Model 1		Model 2		Model 3	
	Disease acceptance		Treatment adherence		Treatment adherence	
	β	t	β	t	β	t
Co: Age	-0.172	-2.870**	-0.047	-0.796	-0.112	-2.02*
Co: Education- Literate	-0.103	-1.294	0.173	2.201*	0.134	1.838
Co: Education- Primary school	-0.002	-0.019	0.118	1.420	0.117	1.526
Co: Education- High school	-0.067	-0.978	0.109	1.605	0.083	1.328
Co: Income less than expense	0.041	0.658	-0.108	-1.746	-0.092	-1.614
Co: Income equals expense	0.143	2.331*	-0.133	-2.189	-0.079	-1.391
Co: Duration of diabetes	-0.037	-0.630	-0.142	-2.389*	-0.157	-2.840**
Co: Diabetes treatment-Oral antidiabetic	0.139	2.124*	-0.044	-0.685	0.008	0.135
Co: Diabetes treatment-Oral antidiabetic &insulin	0.001	0.013	0.011	0.192	0.012	0.213
Co: Chronic diseases other than diabetes	-0.06	-1.110	0.087	1.619	0.064	1.289
X: Social support	0.267	5.162***	-0.413	-8.064***	-0.312	-6.323***
Mi: Disease acceptance					-0.378	-7.387***
F	8.294***		8.978***		14.155***	
R^2^	0.222		0.236		0.347	

Abbreviations: Co, covariate; Mi, mediator; X, independent variable

*p < 0.05, **p < 0.01, ***p < 0.001

**Fig. 1:**
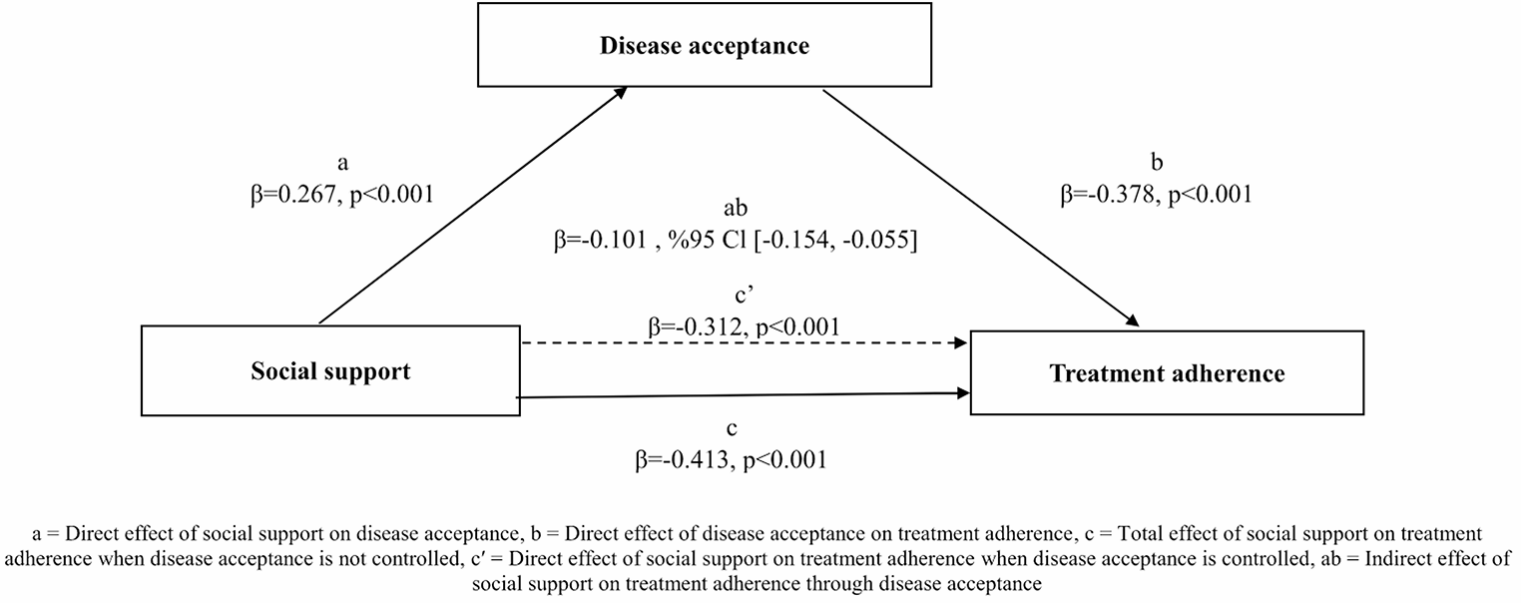
Partial mediation model of disease acceptance in the effect of social support on treatment adherence

Figure 1 shows the partial mediation model demonstrating the role of disease acceptance in the relationship between social support and treatment adherence. The findings reveal that social support has a significant indirect effect on treatment adherence through disease acceptance (β = -0.101, 95% CI [-0.154, -0.055], *p* < 0.001). This indicates that higher levels of social support contribute to improved treatment adherence by enhancing disease acceptance.

## Discussion

This study examined the relationship between social support, disease acceptance and treatment adherence in 332 Turkish adults with type 2 diabetes. The study yielded significant findings on the effects of social support and disease acceptance on adherence to diabetes treatment. The study’s main findings confirmed our hypothesis that social support significantly influences adherence to diabetes treatment and that disease acceptance mediates this relationship. Linear regressions and mediation analysis have shown that higher social support and disease acceptance are associated with better treatment adherence. At the same time, the acceptance of the disease partially mediated the effect of social support on treatment adherence. It has been confirmed that social support can indirectly improve treatment adherence by increasing disease acceptance. Disease acceptance and social support explained a significant variance in treatment adherence.

Social support, which expresses the support perceived and received by the patient from social networks such as family and friends, is also defined as a psychological sense of belonging, acceptance and help that increases the individual’s ability to cope better with stressful conditions (Mohebi et al., 2018; Shao et al., 2017). Spouses, family members, healthcare professionals, relatives, friends, neighbors, colleagues, individuals with the same disease, and even social networks on the Internet can play a role in social support (Mohebi et al., 2018). Social support can encourage people with diabetes to adhere to their treatment plans. In addition to making it easier for people with diabetes to cope with their disease, it can help improve their self-efficacy and the ability to practice self-care behaviours (Bouldin et al., 2017). In particular, family members can contribute to treatment compliance by supporting diabetic patients in subjects such as creating and implementing a healthy diet plan, regular use of medications, exercise and foot care (Mostafavi-Darani et al., 2020; Olagbemide et al., 2021; Onyango et al., 2023; Smalls et al., 2015; Świątoniowska-Lonc et al., 2021). A previous study determined that social support was significantly associated with medication adherence, diet adherence, and foot care, and 11.6% of the variance in medication adherence in diabetic patients was explained by social support (Smalls et al., 2015). In this regard, the findings of the present study confirmed the results of other studies that reported a positive relationship between social support and adherence to treatment in individuals with type 2 diabetes and associated the lack of social support with non-adherence to treatment (Arı & Özdelikara, 2022; Bouldin et al., 2017; Gu et al., 2017; Huang et al., 2021).

Illness acceptance is related to the individual’s adopting a positive attitude towards the disease, recognizing the importance of the disease, encouraging the patient’s ability to cope with the effects of the disease and the lifestyle changes it requires, and facilitating the adaptation process (Brzoza et al., 2022). The individual’s acceptance of the disease contributes to the development of self-efficacy by effectively utilizing their resources and capabilities to manage the disease (Şireci E., 2017). Patients who accept the disease can approach their treatment plan more positively. Denying the disease can lead to poor adherence to treatment. The study determined that the disease’s acceptance in individuals with type 2 diabetes had as much effect as social support on adherence to treatment. Its results confirmed previous studies reporting that disease admission plays a vital role in adherence to treatment in individuals with type 2 diabetes (Can et al., 2020; Özkaptan et al., 2019). The study determined that participants had a moderate level of disease acceptance and that increased disease acceptance improved treatment adherence. Similar to the results of the current study, Can et al. (2020) reported a positive relationship between diabetes acceptance and diet, foot care, and exercise self-care behaviours. Jaworski et al. (2018) determined that individuals with low diabetes acceptance had low adherence to dietary recommendations and did not follow up on regular blood glucose levels. Bonikowska et al. (2021) reported that as the disease’s acceptance level in diabetes patients increased, adherence to treatment recommendations improved, and disease admission explained 9.7% of the variance in adherence to treatment.

The study found that a significant portion of the variance in disease acceptance was explained by social support. Furthermore, disease acceptance was identified as a partial mediator in the relationship between social support and treatment adherence. This finding showed that social support could increase treatment adherence by increasing disease acceptance. Besen and Esen (2012) reported in their study that positive and significant relationships exist between social support and acceptance of the disease in individuals with diabetes. Arı and Özdelikara (2022) determined that as family support increases, acceptance of the disease increases. Although these studies support the results of the current study, studies on the effect of social support on disease acceptance and the role of disease acceptance in the relationship between social support and treatment adherence seem to be limited, and more studies are needed on these issues.

### Limitations

Besides the significant strengths of the study, there are also some limitations. Since the study has a cross-sectional design, it is challenging to establish a definite causal relationship between social support, disease acceptance, and treatment compliance. Because the study was conducted in a single center, the sample may not wholly represent the target group. This may prevent the generalization of the results to the entire type 2 diabetes population. In addition, data collection using only self-report questionnaires may have affected the results by causing reporting bias.

## Conclusion

This study is significant from a clinical practice perspective as it highlights the critical roles of social support and disease acceptance in improving treatment adherence among individuals with type 2 diabetes. The findings suggest that high levels of social support and illness acceptance can improve treatment adherence and that social support may also indirectly enhance treatment adherence by promoting illness acceptance. Healthcare professionals can be key in guiding patients to identify and utilize social support resources effectively. In this context, nurses and other healthcare professionals working with individuals with diabetes can develop specific interventions aimed at strengthening social support networks and encouraging patients to accept their disease. Thus, treatment adherence in individuals with diabetes can be improved, and the risk of long-term diabetes-related complications can be reduced.
